# Acetazolamide Use in the Management of Refractory Acute Decompensated Heart Failure in the ICU

**DOI:** 10.15190/d.2026.2

**Published:** 2026-03-03

**Authors:** Meher Ayyazuddin, Adelyn Mendoza, Vismay Patel, Rubba Shoukat Khan, Rehan Shah

**Affiliations:** ^1^Department of Internal Medicine, Hudson Regional Health Bayonne University Hospital, Bayonne, NJ, USA

**Keywords:** Acute decompensated heart failure (ADHF), congestive heart failure, diuretic resistance, acetazolamide use.

## Abstract

Diuretic resistance is a major therapeutic challenge in acute decompensated heart failure (ADHF). Acetazolamide, a carbonic anhydrase inhibitor, has emerged as a potential adjunct to conventional loop diuretics, as demonstrated in the ADVOR trial. We present a 74-year-old woman with acute coronary syndrome complicated by cardiogenic shock and refractory pulmonary edema despite inotropic support and guideline-directed therapy. The patient received intravenous acetazolamide 500 mg daily for three days, resulting in marked diuresis and radiographic resolution of pulmonary edema within 72 hours. The patient improved clinically. This case supports the potential utility of acetazolamide as an adjunctive strategy for overcoming diuretic resistance in ADHF.

## INTRODUCTION

Congestive heart failure (CHF) is a clinical syndrome resulting from impaired cardiac output and volume overload^1^. Despite advances in guideline-directed medical therapy, 20–30% of patients hospitalized with acute decompensated heart failure (ADHF) develop diuretic resistance. This is associated with prolonged hospitalization and poorer outcomes^2,3^. Acetazolamide inhibits proximal tubular sodium reabsorption, promoting natriuresis when added to loop diuretics^4^. The ADVOR trial demonstrated that acetazolamide, when co-administered with intravenous loop diuretics, improved decongestion rates and shortened hospital stays without increasing adverse renal outcomes^5-7^. Here, we present a patient who demonstrated rapid decongestion after adjunctive acetazolamide use, highlighting its clinical utility.

## CASE PRESENTATION

We present the case of a 74-year-old female with a history of hypertension, chronic obstructive pulmonary disease (COPD), who presented to the emergency department with altered mental status, chest pain, and shortness of breath. Initial electrocardiogram demonstrated ST-segment elevation in leads II, III, and aVF with reciprocal depression in aVL and V2–V3. Serial cardiac biomarkers revealed a rising troponin I trend (0.58 → 77.70 → 234.00 ng/mL).

The patient was admitted for urgent coronary angiography and underwent three percutaneous coronary interventions (PCI): drug-eluting stents were placed in the right coronary artery (day 1) and the left anterior descending artery (day 2), followed by a repeat PCI for mid-RCA stent thrombosis. Intravascular lithotripsy was performed to reduce the heavy calcific burden, followed by high-pressure balloon post-dilatation. She was maintained on dual antiplatelet therapy (aspirin and clopidogrel); however, clopidogrel resistance was suspected, prompting a switch to ticagrelor.

Her post-procedural course was complicated by cardiogenic shock with worsening dyspnea and hypoxemia, necessitating bilevel positive airway pressure (BiPAP) support. The initial transthoracic echocardiogram showed an ejection fraction (EF) of 40–45% with otherwise preserved cardiac structure. Guideline-directed medical therapy (GDMT) was initiated, including a beta-blocker, ACE inhibitor, aspirin, clopidogrel, and a statin. Despite treatment and subsequent transition to 3 L nasal cannula oxygen, she continued to experience chest pain, dyspnea, fatigue, and generalized weakness. Spironolactone was later added. Serial chest radiographs revealed progressive bilateral alveolar infiltrates consistent with pulmonary edema, small bilateral pleural effusions, and cardiomegaly.

The patient’s clinical condition deteriorated further with worsening shortness of breath and somnolence. Repeat echocardiography demonstrated a severely reduced LVEF of 25–30%, extensive regional wall-motion abnormalities, and moderate-to-severe mitral regurgitation. Inotropic support with intravenous milrinone was initiated, while beta-blockers and ACE inhibitors were held due to hypotension. Despite optimal inotropic therapy, her pulmonary edema persisted with minimal symptomatic improvement.

Of note, renal function remained preserved (creatinine 0.8 mg/dL; GFR 119 mL/min/1.73 m²) (**Table 1**).

**Table 1:** Decongestion Parameters During Acetazolamide Therapy

Day	Urine Output (mL/24h)	Net Fluid Balance (mL)	Weight (kg)	Serum HCO₃⁻ (mmol/L)	Creatinine (mg/dL)
Day 0 (Pre-AZT)	1100	+500	71.2	26	0.8
Day 1	3100	-2200	69.8	22	0.8
Day 2	2800	-1900	69.0	21	0.8
Day 3	2500	-1500	68.5	21	0.9

**Figure 1:**
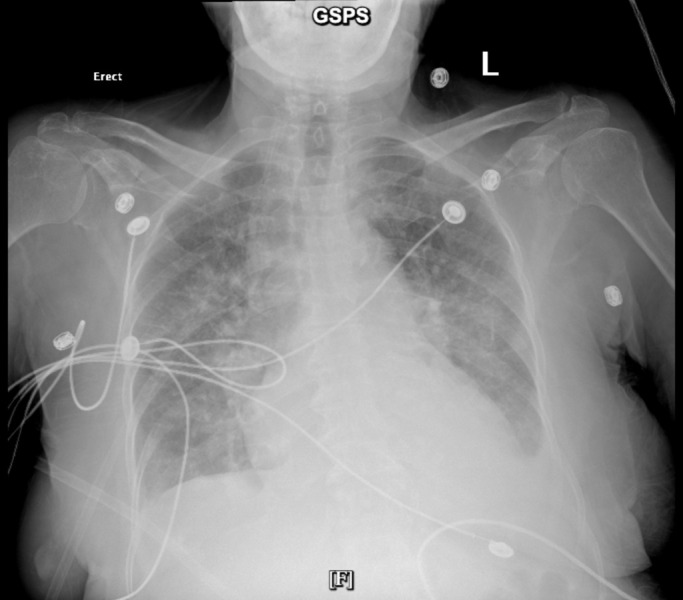
Baseline Chest Xray at initiation of acetazolamide. Portable AP chest radiograph (08/08/2025): Diffuse bilateral perihilar opacities and small pleural effusions consistent with pulmonary edema; cardiomegaly present. Image obtained on hospital day 0, immediately prior to initiation of adjunctive acetazolamide.

**Figure 2:**
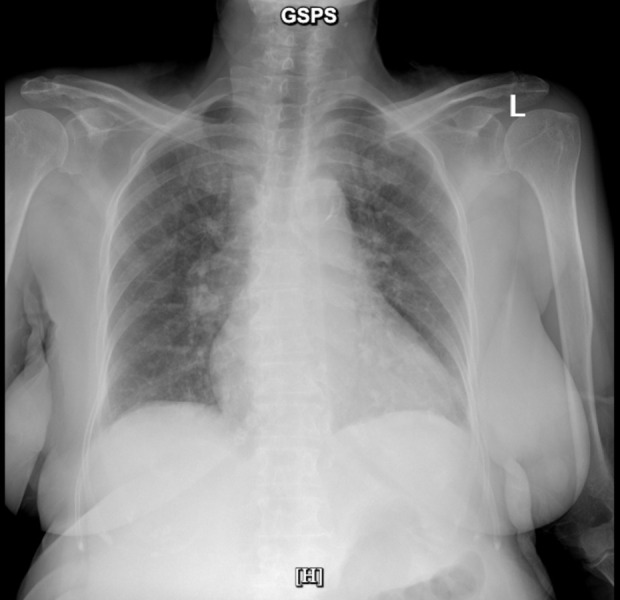
Clinical improvement following adjunctive acetazolamide therapy. Portable AP chest radiograph (08/12/2025): Marked interval clearing of alveolar opacities with resolution of pulmonary edema; cardiomegaly persists. Image obtained on hospital day 4, following three days of adjunctive acetazolamide therapy

Given refractory congestion, a single dose of acetazolamide was administered with close monitoring. Within 72 hours, the patient’s dyspnea and fatigue markedly improved, oral intake resumed, and follow-up chest radiography (**Figure 1** and **Figure 2**) demonstrated substantial resolution of pulmonary edema, though cardiomegaly persisted. A total of three doses of acetazolamide were given, resulting in significant clinical and radiographic improvement. She was successfully weaned off milrinone, transitioned to telemetry, and remained hemodynamically stable on follow-up imaging, which confirmed near-complete resolution of pulmonary congestion.

## DISCUSSION

This case underscores the value of acetazolamide as an adjunctive option in the management of challenging heart failure presentations. Findings from the ADVOR trial demonstrated that adding acetazolamide to loop diuretics enhanced the likelihood of achieving effective decongestion, regardless of baseline renal function. Notably, its benefits on natriuresis and diuresis were most pronounced among patients with reduced estimated glomerular filtration rate (eGFR). Crucially, achieving decongestion remains strongly linked to improved clinical outcomes, even in the setting of worsening renal function (WRF)^8^.

Acetazolamide, a diuretic that blocks proximal tubular sodium reabsorption, enhances natriuresis when added to conventional loop-diuretic therapy. Its use has been shown to accelerate and augment decongestion, while also contributing to a shorter duration of hospitalization^6^. Diuretic resistance is common and arises when natriuresis remains insufficient despite seemingly optimal diuretic dosing, posing one of the most formidable barriers in the management of acute decompensated heart failure. It can be estimated using the BAN-ADHF scores^9^. It not only complicates fluid removal but also signals a trajectory toward worse clinical outcomes^10^. Evidence from recent trials suggests that patients with impaired renal function (eGFR < 40 mL/min/1.73 m²) often require higher loop diuretic doses to achieve decongestion^8^. Yet in contrast, our patient—with preserved kidney function and adequate glomerular filtration - remained refractory to conventional therapy, underscoring the complexity of this phenomenon.

Current Heart Failure Society of America guidelines for ADHF recommend titrating loop diuretics to achieve a rate of diuresis sufficient for optimal volume control^11^. However, evidence indicates that continuous loop-diuretic infusions do not confer advantages over intermittent bolus dosing, as demonstrated in the DOSE trial^12^. More recent randomized studies, including ADVOR and CLOROTIC, have shifted attention toward early sequential nephron blockade, with the addition of agents such as intravenous acetazolamide (500 mg once daily) or thiazide diuretics^13,14^. This raises an important clinical consideration: should acetazolamide be introduced earlier in the treatment algorithm when diuretic resistance is suspected, with the aim of improving both decongestion and hospital length of stay?

In our case, the patient demonstrated marked improvement within three days of initiating acetazolamide, aligning with the primary endpoint reported in the ADVOR trial [8]. Beyond reinforcing trial findings, this case also addresses a notable limitation of existing evidence - ethnic representation. Mullens et al. observed that nearly all ADVOR participants were White, whereas our patient was of South Asian descent^6^. This highlights the importance of validating therapeutic strategies across diverse populations, as genetic, physiologic, and socioeconomic factors may influence treatment response and outcomes. Expanding the evidence base to include underrepresented groups is essential for ensuring that advances in heart failure management are both equitable and broadly applicable.

## LIMITATIONS

This report has several important limitations. First, as a single-patient case report, causal inferences regarding the efficacy of acetazolamide cannot be established, and the observed clinical improvement may have been influenced by unmeasured confounders, concurrent supportive care, or the natural course of decompensation. Secondly, the short duration of follow-up precludes assessment of longer-term outcomes, including rehospitalization, renal trajectory, or mortality. Finally, while this case contributes to diversity in the existing literature, a single observation in an underrepresented ethnic group cannot address broader questions of generalizability. Larger, prospective studies with diverse populations and standardized decongestion metrics are needed to better define the optimal timing, patient selection, and long-term impact of acetazolamide in acute decompensated heart failure.

## CONCLUSION

This case emphasizes the potential of acetazolamide as a valuable adjunct in acute decompensated heart failure, particularly in the setting of diuretic resistance. By mirroring the efficacy observed in clinical trials while extending applicability to a more diverse patient population, it underscores both the therapeutic promise and the need for broader, more inclusive studies. Future research should prioritize representation and explore earlier integration of acetazolamide into treatment algorithms to optimize outcomes in real-world practice.

## Key Points

- *Adjunctive acetazolamide facilitated rapid and effective decongestion in a patient with refractory acute decompensated heart failure despite preserved renal function and ongoing loop-diuretic therapy, with marked clinical, radiographic, and diuretic response within 72 hours.*

- *Findings align with emerging trial evidence from the ADVOR trial supporting early sequential nephron blockade, highlighting acetazolamide as a practical strategy to overcome diuretic resistance, improve natriuresis, and potentially shorten hospitalization without worsening renal function.*

- *This case expands clinical applicability to an underrepresented population and underscores the need for larger, diverse studies to define optimal timing, patient selection, and long-term outcomes of acetazolamide use in contemporary ADHF management.*
